# Definition of network types – Prediction of dough mechanical behaviour under shear by gluten microstructure

**DOI:** 10.1038/s41598-019-41072-w

**Published:** 2019-03-18

**Authors:** Isabelle Lucas, Hannes Petermeier, Thomas Becker, Mario Jekle

**Affiliations:** 1 Technical University of Munich, Institute of Brewing and Beverage Technology, Research Group Cereal Technology and Process Engineering, 85354 Freising, Germany; 2 Technical University of Munich, Chair of Mathematical Modelling of Biological Systems, 85748 Garching, Germany

## Abstract

This study defines network types of wheat gluten to describe spatial arrangements of gluten networks in relation to dough mechanical behaviour. To achieve a high variety in gluten arrangements, ten specific and unspecific gluten-modifying agents in increasing concentrations were added to wheat dough. Gluten microstructure was visualized by confocal laser scanning microscopy and quantified by protein network analysis. Dough rheological behaviour was determined by both oscillatory and creep-recovery tests. Based on correlation matrices and principal component analysis, six different network types were identified and associated to their rheological characteristics: a cleaved (low viscous), rigid (highly viscous), spread (viscoelastic), strengthened (viscoelastic), particulate and dense (highly viscous) or particulate and loose (low viscous) network. Furthermore, rheological dough properties of specifically gluten-modified samples were predicted with five microstructural gluten attributes (lacunarity, branching rate, end-point rate, protein width, average protein length) and assigned properly by the obtained partial least square model with an accuracy up to 90% (e.g., R^2^Y = 0.84 for G*, 0.85 for *tanδ*, 0.90 for J_max_). As a result, rheological properties of wheat doughs were predicted from microstructural investigations. This novel, quantitative definition of the relation between structure and mechanical behaviour can be used for developments of new wheat products with targeted properties.

## Introduction

Due to its unique viscoelastic properties, the gluten network is the most relevant physicochemical structuring component in wheat products. It is responsible for the mechanical behaviour of dough, and it affects the gas holding properties, texture and final bread quality^1,2^. Thus, understanding the relation between gluten network structure and dough functionality makes an optimization of existing products and design of new ones feasible. A lot of research dealt with structure-function relationships of dough so far investigating the gluten network on a microstructural level^1,3–7^ since the analysis of the microstructure is most appropriate to cover the spatial arrangement of gluten proteins. However, relations between dough mechanical behaviour and microstructure were not analysed sufficiently. The reason might be the complex structure of the gluten network and the difficulties in its quantification and interpretation due to a lack of suitable describing attributes.

To overcome this, protein network analysis (PNA), an image analysis for a precise quantification of structural and morphological network attributes of the complex gluten microstructure, was established^8^. Using this method, several studies showed that dough mechanical properties could be elucidated by means of the microstructural attributes of PNA^9–11^. In addition, correlations between protein microstructure and dough mechanical behaviour were demonstrated. Even though the studies mentioned above presented correlations between dough microstructure and rheological properties, these correlations included only individual dough systems, but no universal relationship. Furthermore, the interpretation of the quantitative results of microstructural attributes, especially the effect of changes in these attributes with regard to the spatial arrangement of the whole network, remained challenging. For that reason, we specified microstructural relations as well as network characteristics in general and classified various spatial arrangements in Lucas, *et al*.^12^. Based on this, we proposed five different types of protein networks. An implementation of these results combined with rheological studies would support a precise and universal definition of the relation between structure and mechanical dough behaviour. Moreover, the protein network attributes could be used to predict dough properties.

Thus, the aim of the study was to identify quantitative relations between gluten microstructure and dough rheology in general. To gain universal assertions, a high variety of gluten spatial arrangements was provoked by specific (glutathione, ascorbic acid, potassium bromate, glucose oxidase, transglutaminase, bromelain) as well as unspecific gluten-modifying agents (reduction and increase of hydration level, addition of rapeseed oil and shortening). By applying oscillatory frequency test within the linear-viscoelastic region (LVE) and creep-recovery test in the non-LVE, the elucidation of fundamental rheological relationships as well as relationships between microstructure and rheology in this wide range of different types of dough systems from very low to high viscosity were aspired. To study the dependencies, correlation matrices and principal component analysis (PCA) were used. Prediction models for dough rheology by using microstructural attributes were obtained by partial least square regression (PLS). While our previous research^12^ focused solely on the protein microstructure and the discovery of network arrangements, the current study aims to confirm and further develop the definitions of proposed network types by rheological investigations as well as to define quantitative relations between structure and mechanical dough behaviour by multivariate statistical evaluations.

## Materials and Methods

### Materials

Four different wheat flours (Type 550, Rosenmühle, Landshut, Germany) were used in order to detect microstructural changes independent of the raw material. Flour characteristics were analysed according to AACC international and AACCi methods as well as to the International Association for Cereal Science and Technology (ICC): the moisture (AACCi 44-01), protein content (AACCi 46-16, N × 5.7), ash (ICC 104/1), and falling number (AACCi 56-81). All specifications and characteristics of the four flours (A, B, C, D) are summarized in the previous publication^12^. Commercial wheat flours usually contain 3 mg/kg flour of ascorbic acid, like the flours A and B, whereas flours C and D did not. Glutathione was supplied by VWR International GmbH (Darmstadt, Germany), ascorbic acid by Carl Roth GmbH + Co. KG (Karlsruhe, Germany), transglutaminase (≥1000 units/g) and glucose oxidase (≥1100 units/g) by AB Enzymes GmbH (kindly provided by AB Enzymes GmbH, Darmstadt, Germany), potassium bromate (KBrO_3_) by ThermoFisher GmbH (Karlsruhe, Germany), bromelain (≥3 units/mg protein) and D(+)glucose by Sigma-Aldrich Chemie GmbH (Steiheim, Germany), Rhodamine B by Merck KGaA (Darmstadt, Germany), rapeseed oil by Cargill Oil Packers bvba (Izegem, Belgien) and shortening (ingredients: palm fat, coconut fat, rapeseed oil, water, emulsifier, NaCl, citric acid, aroma, carotin) by MeisterMarken (CSM Deutschland GmbH, Bingen, Germany).

### Dough preparation

Dough preparation was already described in^12^ and is summarized in the following section. All dough samples consisted of wheat flour and demineralized water produced with the required kneading time, water concentration and recipe for each standard wheat dough according to AACCi method 54-70.01, see Table 1. Specific as well as unspecific gluten-modifying agents were added separately to a standard wheat dough in varying concentrations. The targeted resistance of 500 Farinograph units was determined in a Z-kneader (doughLAB; Perten Instruments, Hägersten, Sweden) in reliance on the water absorption and the moisture (corrected to 14%), which is dependent on the flour type (A, B, C, D) as well as on the storage time of each flour. All dough variations were produced in triplicate.

**Table 1:** Variations of produced dough samples.

	Abbreviation	Variation	Concentrations	Flour type	Water addition (mL/100 g flour)	Kneading time* (s) to 500 FU
Specific gluten-modifying agents	ASC	Ascorbic acid	0.0, 25.0, 50.0, 100.0, 150.0 and 200.0 mg/kg flour	C	56.17	300
	BRN	Bromelain	0.0, 200.0 mg/kg flour	D	61.36	240
	GSH	Glutathione	0.0, 7.5, 15.0, 30.0, 45.0, 60.0 and 75.0 mg/100 g flour	B	57.76	180
	GOX	Glucose oxidase	0.0, 20.0, 40.0, 60.0, 100.0 and 150.0 mg/kg flour, addition of 0.5 g glucose/100 g to each sample	D	61.06	250
	KBrO_3_	Potassium bromate	0.0, 60.0, 120.0 and 180.0 mg/kg flour	D	61.36	240
	TG	Transglutaminase	0.0, 100.0, 1000.0, 2000.0, 5000.0 and 10000.0 mg/kg flour	D	60.49	250
Unspecific gluten-modifying agents	IHL	Increase of hydration level (compared to the standard)	59.18 (standard), 64.87, 69.86, 74.85, 79.84 and 89.82 mL water/100 g flour	A	59.18	180
	RHL	Reduced hydration level (compared to the standard)	57.83 (standard), 53.84, 49.84, 47.58 and 45.85 mL water/100 g flour	B	57.83	180
	ROI	Rapeseed oil	0.0, 5.0, 10.0, 20.0 and 50.0 g/100 g flour	B	58.32	180
	SHO	Shortening	0.0, 5.0, 10.0, 15.0, 20.0 and 50.0 g/100 g flour	B	58.32	180

Specific as well as unspecific gluten-modifying agents were added to standard wheat doughs in varying concentrations. A standard wheat dough consists of flour and demineralized water. *Kneading times were rounded to the nearest tens.

### Rheological analysis

An AR-G2 rheometer (TA instruments, New Castle, USA) with parallel cross-hatched plates (Ø 4.0 cm) to prevent slipping, a constant gap of 2.0 mm and a smart swap Peltier plate temperature system (30 °C constant temperature during measurement) were used to determine the viscoelastic properties of each dough sample. For this purpose, oscillatory frequency sweep and creep-recovery tests were performed as reported by Bernklau, *et al*.^8^.

#### Oscillatory frequency sweep test

The oscillatory frequency sweep was performed within the LVE, which was determined before with an amplitude sweep test. Oscillatory frequency tests were carried out at a constant deformation of 0.1% in a frequency range of 0.1 to 50 Hz. Results were evaluated by the complex shear modulus G* (Pa) at 1 Hz and the loss factor *tanδ* (−) (the ratio of the loss modulus *G″* and storage modulus *G′*). In addition, the complex shear moduli (at 0.1–50 Hz) were fitted according to the power law equation in order to obtain distinct information about network characteristics:

1 $${G}^{\ast }(\omega )={A}_{f}{\omega }^{1/z}$$

Where *ω* (s^−1^) is the angular frequency. *A*_*f*_ (Pa s^1/z^) is calculated by the model power law, G* values over the whole frequency period 0.1–50 Hz. *A*_*f*_ is interpreted as the network strength (strength of interactions between the network components) and *z* (−) as the network connectivity (extend of interactions)^13,14^.

#### Creep-recovery test

During dough processing, dough is exposed to large strains and shear rates, which represent a mostly nonlinear viscoelastic behaviour. This is why the creep-recovery test was performed in the non-LVE region, as recommended by Van Bockstaele, *et al*.^15^. A constant shear stress of 250 Pa for 180 s and a relaxation time of 360 s was applied. Results were evaluated by the creep compliance *J*_*max*_ (Pa^−1^) (maximal compliance at the end of the creep phase), creep-recovery compliance *J*_*r*_ (Pa^−1^) (minimal compliance at the end of the recovery phase) and the relative elastic part *J*_*el*_ (%) (*J*_*r*_*/J*_*max*_ * 100). In order to gain further information about the dough rheological behaviour, especially the instantaneous change in compliance and viscous deformation, creep data were analysed with a four parameter (Eq. 2) and recovery data with a five parameter Burgers model (Eq. 3) according to Van Bockstaele, *et al*.^15^:

2 $${J}_{creep}(t)={J}_{0creep}+{J}_{1creep}[1-\exp (-\,t/{\lambda }_{creep})]+t/{\eta }_{0}$$

3 $${J}_{rec}(t)={J}_{0rec}+{J}_{1rec}[1-\exp (\,-\,\,t/{\lambda }_{1rec}\,)]+{J}_{2rec}[1-\exp (\,-\,\,t/{\lambda }_{2rec}\,)]$$

With the time *t*, *J*_0_ (Pa^−1^) the instantaneous compliance (pure elastic part), *J*_1_ (Pa^−1^) and *J*_2_ (Pa^−1^) the retarded elastic compliances, *λ*_1_ (s) and *λ*_2_ (s) the retardation times and *η*_0_ (Pa s) the steady state viscosity. A five parameter Burgers model with two Kelvin elements was chosen for the recovery phase due to a better representation of the experimental data^16^.

### Microstructure analysis

Microstructure analysis has been carried out in Lucas, *et al*.^12^ and summarized as follows.

#### Confocal laser scanning microscopy (CLSM)

For the visualization of the gluten proteins by CLSM, all dough samples were stained with Rhodamine B (0.01 g/100 mL water) according to the bulk water technique^17^. An eclipse Ti-U inverted microscope with an e-C1 plus confocal system (Nikon GmbH, Düsseldorf, Germany) with a Plan Apo VC 60x/1.40 oil objective and a 534 nm laser (emission 590/50 nm) were used for microstructure analysis. Eight independent (non-overlapping) images were taken of each sample with a resolution of 1024 × 1024 pixel and a size of 215 × 215 µm.

#### Protein network analysis

On CLSM micrographs, image analysis quantified network characteristics using the method protein network analysis (PNA)^8^. For this purpose, the software AngioTool64 version 0.6a (National Cancer Institute, National Institute of Health, Maryland, USA) was used^18^. The settings and calibrations were adjusted as described by Lucas, *et al*.^12^. The following network attributes were evaluated: the structural network attributes branching rate (describes the network connectivity), end-point rate (describes the weakness of a network), average protein length (length of a continuous protein particle) and protein width (thickness of protein threads) as well as the morphological attribute lacunarity (attribute for the amount and size of network gaps; describes irregularities of a structure).

### Statistical analysis

Multivariate statistics, principal component analysis (PCA) and partial least squares (PLS) analysis were performed with JMP Pro software (version 12.2.0, SAS Institute Inc., Cary, NC, USA). The goodness of fit of linear and nonlinear regressions were represented by R^2^.

PCA was used to identify structural similarities or clusters based on rheological and microstructural characteristics. PCA was performed for solely rheological data as well as for rheological combined with microstructural data (for all samples as well as for solely gluten-modified samples). Evaluation was performed by score (matrix of scatterplots of the scores for pairs of principal components) and loading plots (matrix of two-dimensional representation of factor loadings)^19^.

For PLS model, the response variable Y (here: rheological attributes) was explained by several predictor variables X characterizing the microstructural attributes to determine and quantify the relation between them. By introducing so-called latent variables based on linear combinations of the original data, PLS identifies the factors, which explain the highest variance between the predictors and the response. PLS can deal with multicollinearity amongst the data as well as a large number of X variables^20–22^. PLS models were estimated with the NIPALS (nonlinear iterative partial least squares) algorithm with “leave-one-out” cross-validation for model validation. The appropriate number of factors were identified by the minimum Root Mean PRESS (predicted residual error sum of squares). Variable importance for projection (VIP) scores greater 0.8 defined those variables which are relevant indicators and important predictors for the model^19^. VIP were used to point out the microstructural variables with the highest contribution for the projection model of each rheological attribute.

Since some of the data sets showed nonlinear relationships between X and Y values and PLS is based on linear dependency, the response (*Y*) variables (*J*_*max*_, *J*_*r*_, *η*_0_, G*, *A*_*f*_) were linearized as suggested by Wold, *et al*.^23^. For *J*_*max*_, *J*_*r*_ and *η*_0_ the logarithm and for G* as well as *A*_*f*_ the reciprocals were taken.

## Results and Discussion

All ten dough variations, specifically as well as unspecifically gluten-modified, were evaluated collectively to identify relations in general. Initially, appropriate rheological tests and attributes were evaluated for further investigations and correlation to microstructure. Subsequently, the relation between dough rheology and gluten microstructure was investigated first with both unspecifically and specifically-gluten modified samples and second, with solely the specific modified samples. Concluding, the definition of network types is presented. Discussion focusses on different network arrangements and the effect on dough rheology considering all data collectively. For a detailed discussion about the single chemical or technological effects of each specific or unspecific-gluten modifying agents, the authors refer to Lucas, *et al*.^12^.

### Rheological consideration

Generally, the mechanical behaviour is often determined with rheological methods assuming a linear dependency of the viscoelasticity. However, dough mechanical properties during dough processing should be determined in large strains, i.e. out of the LVE^15,24^. Indeed, it is discussed in literature that shear tests within the LVE, like oscillatory measurements, are not sufficient to describe dough properties during processing or to detect differences in doughs of various wheat flour types^24,25^. On the contrary, such methods are widely used to describe dough mechanical behaviour and their suitability is reported as well^26–28^. Thus, shear methods for both measurement ranges, namely oscillatory frequency test in small strain (measured in the LVE) and creep-recovery test in large strains (measured in the non-LVE), were applied in order to evaluate dependencies among both as well as the applicability to characterize dough properties.

**Figure 1:**
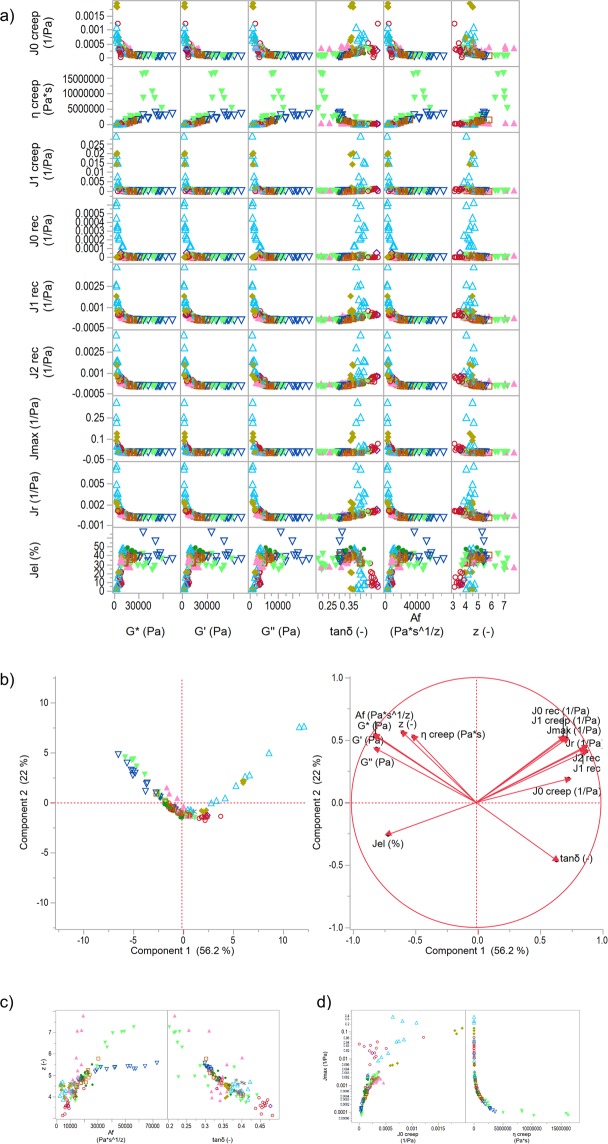
Correlation matrix and principal component analysis of the rheological data of unspecifically as well as specifically gluten-modified samples. (**a**) Correlation matrix of selected rheological attributes (oscillatory frequency test and creep-recovery test) of all gluten-modified samples. (**b**) PCA score and loading plot for the first and second principal component. (**c**) Correlations between z and A_f_ as well as tanδ. (**d**) Correlations between J_max_ and J_0 creep_ as well as η_0_ (J_max_ is presented with log scale). Symbols:  ASC,  KBrO_3_,  BRN,  SHO,  GSH,  GOX,  ROI,  IHL,  RHL,  TG.

Figure 1a,c,d presents the correlation of selected rheological attributes. The correlation matrix containing all attributes can be found in the Supplementary Data 1. Results revealed that most attributes of oscillatory frequency test (G*, *G*′, *G*″, *A*_*f*_) correlated nonlinearly (exponential, R² between 0.66 and 0.81) with those of the creep-recovery test (*J*_*max*_, *J*_*r*_, *J*_*el*_, *J*_0*creep*_, *J*_1*creep*_, *J*_0*rec*_, *J*_1*rec*_, *J*_2*rec*_). This implicates, that despite the large deformations, there are intrinsic properties of the dough matrix, which correspond to the dough behaviour under small strain. Similar results were reported by Van Bockstaele, *et al*.^28^ between creep-recovery and dynamic oscillation characteristics of 17 wheat cultivars highlighting that rheological properties of small deformation tests can be related with nonlinear correlations to large deformation ones. In the current study, also a linear relation between results of the creep-recovery and oscillatory frequency test was found. The steady state viscosity *η*_0_ correlated linearly with the stiffness G* (R² = 0.79) for almost all samples, excluding samples with higher transglutaminase (TG) concentrations than 5000 mg/kg flour (c.f. Fig. 1a) which can be explained by the fact that highly concentrated TG samples showed a much higher viscosity than stiffness, maybe due to the densely clustered protein agglomerates^12^. Hence, for samples with low and moderate TG concentration, the proportionality – the higher the viscosity, the higher dough stiffness – holds. Relations between the different attributes of creep-recovery and oscillatory frequency test were also visible in the loading and score plots of the principal component analysis (Fig. 1b). The score plot confirms the indication of non-linear relations and the loading plot highlights the detected linear relations of some rheological attributes of the correlation matrix (*η*_0_ with attributes of oscillatory frequency test). The score plot showed a separation of the data to stiffer and softer doughs. Oscillatory frequency attributes as well as *η*_0_ characterized RHL (reduced hydration level) and TG samples (stiffer doughs). Conversely, softer doughs, such as samples with increasing water (IHL) or rapeseed oil (ROI) concentration, were characterized by creep-recovery attributes due to a higher deformation.

Focusing on the oscillatory frequency test, the network strength *A*_*f*_ (recorded over whole frequency period 0.1–50 Hz) gives no more distinct information than the variable G* (single value recorded at 1 Hz). Both correlated highly linearly with a R² of nearly 1 (c.f. Supplementary Data). The correlation of the network strength *A*_*f*_ and network connectivity z showed that for larger z values (>5), *A*_*f*_ can take low (SHO), medium (TG) and large values (RHL). This indicates that the network connectivity separates stiff dough samples with different protein network formations. This important fact for the relationship between the rheology and the microstructure will be discussed in detail later (following chapter). Interestingly, the network connectivity factor z correlated inversely proportional with *tanδ* (R² = 0.71), meaning, the lower the network connectivity, the more does the samples act as an (ideal) viscous liquid (c.f. Fig. 1c).

Considering the various variables of the creep-recovery test, it became apparent that most attributes of the Burgers Model (for creep as well as recovery) provide no additional information about rheological behaviour in this experimental setup. The reason is that the common attributes *J*_*max*_, *J*_*r*_ and *J*_*el*_ as well as *J*_*r*_, *J*_1*rec*_ and *J*_2*rec*_ are correlated linearly among each other (R² between 0.90 and 0.97) as well as *J*_*max*_ with *J*_1*creep*_ (R² = 0.68) (c.f. Supplementary Data). However, the Burgers attributes *J*_0 *creep*_ and *η*_0_ were able to discriminate microstructures. The instantaneous compliance *J*_0*creep*_, which describes the pure elastic part of a dough sample, increases with higher dough deformation (*J*_*max*_). However, there is a shift to a nonlinear extent for dough samples with a high creep compliance *J*_*max*_ (GLU, high IHL samples) to comparatively very low *J*_0*creep*_ values. Very low values for the relative elastic part *J*_*el*_ characterized these samples. As illustrated in Fig. 1d, *η*_0_ correlated nonlinearly (exponential) with all compliances (e.g., R² = 0.97 for *J*_*max*_). This indicates that dough samples with an extremely high deformation *J*_*max*_ (GLU, ROI or IHL samples) correspond with a low steady state viscosity *η*_0_, whereas low deformed doughs (TG > 1000 mg/kg flour, RHL) are characterized by a very high viscosity.

As clearly shown, correlations revealed strong dependencies between rheological properties measured with small and large deformation tests. Since most relations were exponential, an evaluation of both methods is important when taking into account that dough rheological behaviour is related to the final product properties and for getting insights of structure-function relationships. Although the relevance of those measurements for conclusions on dough functionality and final product properties is discussed contradictory in literature^24^, there are studies showing that e.g., viscoelastic data gained by creep-recovery measurements correlate linearly with empirical rheological parameters (dough extensibility, mixing time) and bread volume^29^. As well, other studies reported that G* correlated nonlinearly^28^ and *J*_*max*_^30^ as well as *J*_*r*_^31^ linearly with bread volume. For a comprehensive characterisation of wheat dough mechanical behaviour related to processing performance and product properties, further rheological analysis such as biaxial extensional measurements or even under hyperbolic contraction flow is recommended, since biaxial forces occur during proofing and baking^24,32,33^. However, the main focus of this study was to define relations between gluten microstructure and mechanical behaviour of dough at all and to start with shear measurements. Thus, biaxial investigations should be considered in future studies. In the current study, the mechanical behaviour under shear was focussed.

Hence, most relevant information about dough rheological behaviour regarding the aims of this study are provided by the attributes *J*_*max*_, *J*_*r*_, *J*_*el*_, *η*_0_, G*, *tanδ*, *A*_*f*_ and *z*. Thus, these attributes were considered for further investigations in the following sections.

### The link between rheology and microstructure – Unspecifically- combined with specifically-gluten modified samples

#### Correlation matrix and PCA

**Figure 2:**
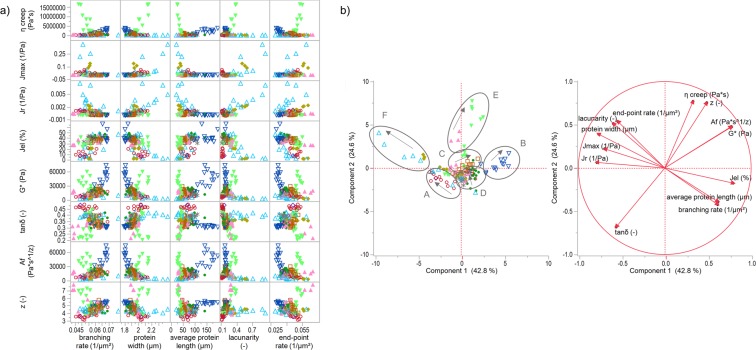
Correlation matrix and principal component analysis of unspecifically as well as specifically gluten-modified samples. (**a**) Correlation matrix of rheological and microstructural attributes of all gluten-modified samples. (**b**) PCA score and loading plot for the first and second principal component. Labelled clusters were used for the identification of different network types. Arrows within the clusters denote increasing concentrations. Symbols:  ASC,  KBrO_3_,  BRN,  SHO,  GSH,  GOX,  ROI,  IHL,  RHL,  TG.

Figure 2a shows the correlation matrix of microstructural versus rheological attributes. At first, no clear linear relations between those attributes were found. A closer observation of the data revealed some exponential relations of G*, *A*_*f*_ or *J*_*el*_ with the five microstructural attributes on some subsets of the data (except TG samples), especially the IHL and RHL samples, which represent unspecifically gluten-modified samples by reducing or increasing water concentrations. Therefore, principal component analysis was performed to identify relations or groupings within the data set. While PCA of solely the rheological properties (section “Rheological consideration”) separated the data to softer and firmer doughs, PCA of rheological combined with microstructural attributes (Fig. 2b) highlighted several more clustered data. One grouping, labelled with “E”, was represented by samples with a TG concentration of 2000 mg/kg flour and above, and by SHO samples ≥50 g/100 g flour. These samples were most represented by the second component, which is dominated by the steady state viscosity *η*_0_ (increase) and inversely by the loss factor *tanδ* (decrease). These are very stiff doughs with a behaviour like an elastic solid of a high resistance to deformation. Note that according to microstructure analysis^12^, these samples were characterized by clustered protein particles, which were scattered densely. These unevenly distributed protein networks with high TG concentrations were confirmed by Autio, *et al*.^34^. Since even highly different sample types, modified specifically with the enzyme transglutaminase (TG) and unspecifically with shortening (SHO), showed the same microstructural and rheological characteristics, it can be concluded: gluten network arrangement of densely clustered agglomerates are related to highly viscous and stiff wheat doughs.

Dough samples with reduced water concentrations compared to a standard dough (RHL samples) formed a further cluster in PCA score plot (labelled with “B” in Fig. 2b). Especially the high dough firmness, defined by G* and the network strength *A*_*f*_, affected the separation in the score plot. Furthermore, as already indicated in section “Rheological consideration”, network connectivity *z* separated wheat doughs with a high network strength *A*_*f*_. The network connectivity, which describes the extent of interactions of involved units, was 17–37% lower than for cluster E for almost the same network strength. In contrast to cluster E (high SHO and TG samples), samples of cluster B had a larger relative elastic recovery by an average of 25%. These effects led to the conclusion that doughs of cluster B show higher elastic behaviour than those of cluster E even if the remaining rheological attributes seemed to be quite similar. However, gluten network of cluster B samples were completely different. These samples had the highest branching rate as well as average protein lengths and lowest end-point rates compared to all other dough samples, forming a continuous and uniform network structure.

As visualized in the PCA score plot (Fig. 2b) and the discussion from the rheological point of view already indicated, samples with increasing water (IHL) or rapeseed oil (ROI) concentration formed a cluster (labelled with “F”). Those doughs can be characterized by clustered agglomerates, but in contrast to doughs of the cluster E, widely scattered (very high values for lacunarity) due to a dilution and plasticizing effect of the additives. This kind of network results in a low resistance to deformation (high *J*_*max*_, low *η*_0_) and a very low relative elastic recovery (*J*_*el*_) of dough. For a better understanding, schematic illustrations of different network types based on the detected PCA clusters are visualized in the section “Definition of gluten network types”.

Residual data were accumulated around the centre of the PCA score plot. Further clusters can be detected within the data (A, C, D), however, mainly represented by specifically gluten-modified samples. Hence, a further investigation about microstructural and dough rheological relationships was done with solely specifically gluten-modified samples in the following sections.

#### Prediction models by means of PLS analysis

An aim of this study was to determine statistical models to predict dough rheology by microstructural attributes. Since no distinct relations were detected in the correlation matrix above, also no significant prediction models were found with PLS analysis. Although the rate of explained variation of *Y* by *J*_*max*_ or *J*_*r*_ were about 60%, IHL values dominated the predictions pretending an appropriate fit. Residual by predicted plot revealed clearly that the model did not fit the data (data not shown).

### The link between rheology and microstructure – Specifically gluten-modified samples

Concluded from the results above, the correlations between dough rheological and gluten microstructural attributes were highly predominant of unspecifically gluten-modified samples, especially IHL and RHL samples. Furthermore, not only the gluten network was affected by unspecific gluten-modifying agents, but also the whole dough system was influenced by plasticizing and dilution effects and thus, the dough rheology. Hence, a sole elucidation of the relation between dough rheology and gluten microstructure can only be determined with correlation and PCA analysis of solely specifically gluten-modified samples.

#### Correlation matrix and PCA

**Figure 3:**
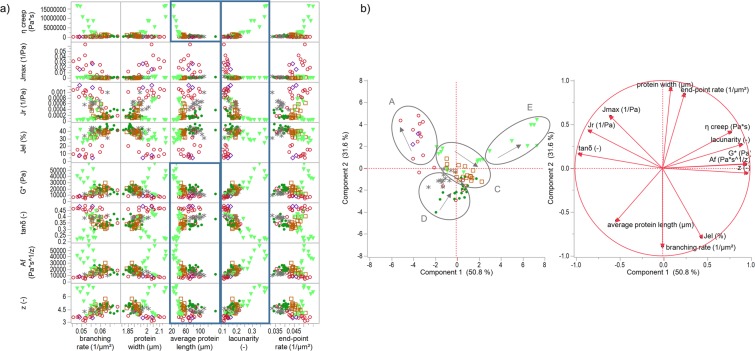
Correlation matrix and principal component analysis of specifically gluten-modified samples. (**a**) Correlation matrix of rheological and microstructural attributes of specific gluten-modified samples. Key correlations are denoted with a blue rectangle. (**b**) PCA score and loading plot for the first and second principal component. Labelled clusters were used for the identification of different network types. Arrows within the clusters denote increasing concentrations. Symbols  ASC,  KBrO_3_,  BRN,  GSH,  GOX,  TG.

In contrast to the correlation matrix of all samples, the correlation of specifically gluten-modified samples revealed distinct correlations between the morphological attribute lacunarity with the rheological attributes. As visualized in Fig. 3a, the lacunarity showed exponential dependency $y=a+b{e}^{cx}$(e.g., R² = 0.91 for *η*_0_) with the attributes of creep-recovery test and linear correlations (e.g., R² = 0.70 for G*) with those of the oscillatory frequency test. The network attribute average protein length showed some nonlinear (exponential) correlations as well (R² = 0.93 for *η*_0_), although some correlations were not significant (e.g., R² = 0.47 for *A*_*f*_). These correlations indicate, that the higher the lacunarity and the lower the average protein length (length of an interconnected protein particle), the higher the dough stiffness (G*) and the lower dough deformation under strain (*J*_*max*_). However, no direct link between the other network attributes and rheological behaviour was found. Therefore, PCA was performed to detect relations between the attributes or groupings. Figure 3b shows a clear separation between the different clusters in accordance to the protein network classifications of Lucas, *et al*.^12^. Cluster E, predominant of high TG samples, was already detected and described in the previous section. A further cluster is represented by GLU and BRN samples (cluster A), which were specified by a very low lacunarity, low branching-rates and very high end-point rates. These structural characteristics of a weak network are linked to a high dough deformation against strain (*J*_*max*_), a very low relative elastic recovery (*J*_*el*_) and a low dough stiffness (G*). The decrease of dough firmness with GSH addition is in accordance with other studies^27,35^. Cluster D (mainly ASC and KBrO_3_ samples) is mainly influenced by the first principal component, especially high average protein lengths and high protein widths. This kind of network with strengthened protein threads goes along with high values for *J*_*el*_ and *tanδ*. With increasing concentration of the added agents (highlighted with arrows in Fig. 3b), the protein width increased whereas the branching rate decreased. In contrast, gluten networks of cluster C altered conversely with increasing concentration (GOX samples, TG ≤ 1000 mg/kg flour). These networks consist of highly cross-linked and homogenously distributed proteins^12^. Steffolani, *et al*.^36^ reported similar microstructural properties of a continuous, closed and stronger network structure with GOX and TG addition. Rheological properties of cluster C samples showed a viscoelastic behaviour similar to those of cluster D. However, doughs of cluster C were a bit stiffer (G*) and had a higher viscosity (*η*_0_). Since cluster C is allocated around the centre of the PCA score plot, those samples had no extrema in rheological or microstructural properties and can thus be described as regular (standard) up to strengthened doughs.

#### Prediction models by means of PLS analysis

**Figure 4:**
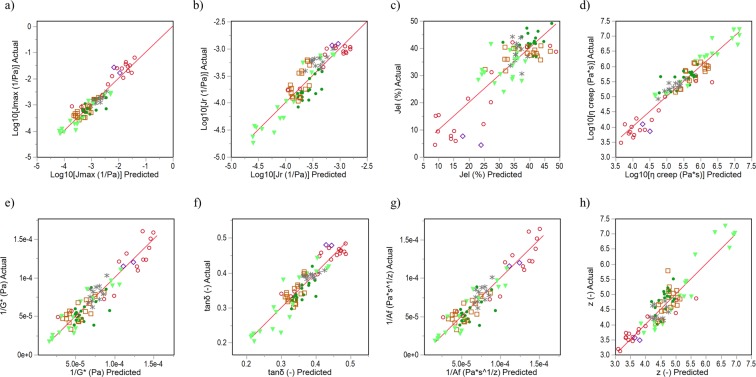
Actual by predicted plots. Rheological attributes were predicted with PLS models of microstructural attributes. Actual by predicted plots are shown for (**a**) log_10_(J_max_), (**b**) log_10_(J_r_), (**c**) J_el_, (**d**) log_10_(η_0_), (**e**) 1/G*, (**f**) tanδ, (**g**) 1/A_f_ and (**h**) z. Symbols  ASC,  KBrO_3_,  BRN,  GSH,  GOX,  TG.

Partial least square regressions were applied to determine prediction models for dough rheology by using microstructural attributes. Since scatterplots suggested some nonlinear dependencies between rheological and microstructural attributes, the suitable transformations of the data resulted in linear relations as described in the section “Statistical analysis”. Table 2 summarizes prediction formula for each rheological attribute, the percentage, which explains the variation for predicted values (R²Y) and Fig. 4 displays the parity-plot. The prediction models clearly show a mathematical relation between dough rheology and gluten microstructure. As the correlation analysis above suggested, rheological attributes of the creep-recovery test depend logarithmically on protein network attributes in the prediction models, whereas those of the oscillatory frequency test showed linear or reciprocal behaviour. R²Y shows, that the logarithmic values of *J*_*max*_ and *η*_0_ explain almost 90% of the variation (c.f. Table 2). The residual rheological attributes were predicted with an R²Y between 72 to 85%. This is remarkably high, since the models are derived from microscopic images, which usually have a higher variance due to operator influences. Furthermore, the models listed in Table 2 predicted rheological properties of standard doughs as well as of the lowest concentration of the unspecific gluten-modified samples ROI, SHO, and RHL. Standard errors of the original value accumulated between 8 and 16% for *J*_*el*_, *tanδ* and *z* values, between 10 to 16% for the logarithmic attributes *J*_*max*_, *J*_*r*_ and *η*_0_, and around 26% for the reciprocal attributes G* and *A*_*f*_.

**Table 2:** Prediction models for selected rheological attributes.

Rheological Attribute	Prediction Formula	PLS factors	VIP	R²Y (%)
Log_10_(**J**_**max**_)	$1.598pw-48.694br+0.016apl-9.188ly+52.705epr-4.874$	4	apl ly	89.65
log_10_(**J**_**r**_)	$0.950pw-29.975br+0.005apl-6.651ly+3.072epr-2.905$	3	apl ly	81.56
**J**_**el**_	$-\,33.843pw+689.778br-0.265apl+72.428ly-1591.011epr+132.938$	4	pw br apl ly epr	71.85
Log_10_(**η**_**0**_)	$57.529br-1.567pw-0.018apl+11.776ly-54.449epr+6.519$	4	apl ly	89.68
1/G*	$9.275\cdot {10}^{-5}pw-2.446\cdot {10}^{-3}br+8.503\cdot {10}^{-7}apl-4.547\cdot {10}^{-4}ly+3.391\cdot {10}^{-3}epr-8.588\cdot {10}^{-5}$	5	br ly	84.26
**tanδ**	$0.151pw-4.067br+7.868\cdot {10}^{-4}apl-1.010ly+1.501epr+0.377$	3	aplly	84.83
1/**A**_**f**_	$9.161\cdot {10}^{-5}pw-2.491\cdot {10}^{-3}br+8.597\cdot {10}^{-7}apl-4.586\cdot {10}^{-4}ly+3.489\cdot {10}^{-3}epr-8.525\cdot {10}^{-5}$	5	pw br ly	83.81
**z**	$2.222pw+81.507br+-\,5.997\cdot {10}^{-3}apl+13.389ly-29.568epr-5.274$	4	apl ly	83.26

Equations for rheology prediction were determined by PLS models of the microstructural attributes protein width (pw), branching rate (br), average protein length (apl), lacunarity (ly) and end-point rate (epr). The models’ dominated attributes are given by VIP (variable importance for projection) values greater than 0.8. R²Y describes the percentage explained for cumulative Y.

Considering the VIPs, prediction models were mainly affected by the morphological attribute lacunarity. In addition, the network attributes average protein length and branching rate had a high impact on the prediction models as well. However, all five network attributes were required for a precise prediction model.

Therefore, in case of wheat doughs with specifically modifying gluten protein additives, the models predict the rheological properties by gluten network attributes very well. This holds too for additives, which modify unspecifically or to a low extent. Hence, it can be deduced that gluten proteins dominate rheological dough properties, even though there are several further influencing factors. It should be noted that some of the prediction models are based on logarithmic relations as well as the deviations (*J*_*max*_, *J*_*r*_, *η*_0_). The predicted values of the sole rheological values remained a higher variation due to an increase of the deviations. Nevertheless, plots of predicted vs. actual values showed promising linear correlations of *J*_*max*_ (R² = 0.8), J_r_ (R² = 0.71) and η_0_ (R² = 0.77).

### Definition of gluten network types

Based on the findings of the sections above, protein microstructure can be classified into six main network types, as summarized in Table 3. Network types correspond to the clusters visualized in Fig. 2 as follows: A – cleaved network, B – rigid network, C – spread network, D – strengthened network, E – particulate, dense network, and D – particulate, loose network. Microstructural evaluation already indicated five network types^12^. Combined with rheological considerations, a sixth network type (rigid network) was identified and an improvement as well as further development of network characteristics related to dough mechanical behaviour for all network types was established in this study. The classification of the network types was performed according to the attribute lacunarity. In contrast to the structural network attributes, lacunarity is a morphological attribute, which pointed out distinct gluten network properties. Furthermore, Gao, *et al*.^11^ proposed the attribute lacunarity as an indicator for gluten network formation and thus, for dough mixing properties and wheat quality, highlighting the importance of this attribute. The lacunarity values are dependent on the resolution of the images. In this study, images were recorded with 1024 × 1024 pixel, which is a commonly used resolution. Since lacunarity is barely dependent on the used magnification in contrast to the residual network attributes^8^, it is an appropriate value for the classification of most dough samples prepared without yeast. Please note that the lacunarity ranges shown in Table 3 should give an indication and are no fixed values. The lacunarity values (which describe holes and gaps within a network) will increase if another component, e.g., gas due to yeast addition, is present. In this case, Table 3 is still applicable for network classifications when comparing the alterations of values to a standard dough as explained subsequently.

**Table 3:** Definition of gluten network types.

	Cleaved network	Rigid network	Spread network	Strengthened network	Particulate, dense network	Particulate, loose network
***Classification by Lacunarity*** (−)	↓ 0–0.16	↓ 0.17–0.26	→ 0.17–0.26	↑ 0.17–0.26	↑ 0.27–0.5	↑↑ >0.5
Branching rate (µm^−2^)	● ↓↓	●●●● ↑↑	●●● ↑↑	●● ↓	● ↓	● ↓↓
Protein width (µm)	●●● ↑	● ↓	●● ↓	●●● ↑	●●● ↑	●●●● ↑↑
Average protein length (µm)	●● ↓	●●●● ↑↑	●● ↑	●●● ↓	● ↓↓	● ↓↓
End-point rate (µm^−2^)	●●●● ↑↑	● ↓↓	●●● ↓	●● ↑	●●●● ↑↑	●●●● ↑↑
***Network description***	Ruptured protein threads, short protein segments	Uniform, dense and continuous structure, highly branched	Homogenous, very branched, elongated and distributed protein threads	Locally strengthened protein threads, continuous	Clustered agglomerates, densely arranged	Clustered agglomerates, widely scattered
***Rheological characteristics***	Low viscous	Highly viscous	Viscoelastic	Viscoelastic	Highly viscous	Low viscous
η_0_ (Pa*s)	●	●●●	●●●	●●	●●●●	●
G* (Pa)	●	●●●●	●●●	●●	●●●●	●
z (−)	●	●●●	●●●	●●	●●●●	●●
tanδ (−)	●●●●	●●	●●	●●●	●	●●
J_el_ (−)	●	●●●	●●●	●●●	●●	●
J_0creep_ (1/Pa)	●●	●	●	●●	●	●●●
J_1creep_, J_1rec_, J_2rec_, J_max_, J_r_ (1/Pa)	●●●	●	●	●●	●	●●●●
Correspond to labels in Fig. 2	A	B	C	D	E	F
Correspond to network type of Lucas, *et al*.^12^	I		II	III	IV	V
***Examples***	GLU ≥ 30 mg/100 g flour, BRN ≤ 200 mg/kg flour	RHL ≤ 49.8 ml/100 g flour	GOX ≥ 40 mg/kg flour, TG ≤ 1000 mg/kg flour	ASC ≥ 50 mg/kg flour, KBrO_3_ ≥ 60 mg/kg flour SHO ≤ 15 g/100 g flour	TG > 1000 mg/kg flour, SHO ≥ 50 g/100 g flour	IHL ≥ 69.9 ml/100 g flour, ROI ≥ 50 g/100 g flour

The classification is performed according to the microstructural attribute lacunarity (determined on images with a resolution of 1024 × 1024 pixel and size of 215 × 2015 µm). Network attributes are described by their strength ● (low) - ●●●● (very high). Arrows (↓→↑) denote the development of microstructural attributes of a network type**’**s typical sample compared to its corresponding standard wheat dough.

Table 3 supports the interpretation of protein network micrographs in future studies. Evaluation needs to be performed in comparison to a standard dough (without any additive) to distinguish structural alterations. It is created to use it as a guiding classification scheme according to the following procedure:

- Determination of lacunarity range
- Comparison of PNA results of the sample with corresponding standard dough (without any additive) regarding to an increase (↑) or decrease (↓) of the values
- Identification of the corresponding network type

**Figure 5:**
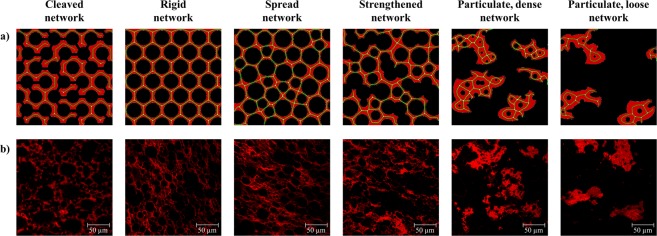
Illustrations of gluten network types. (**a**) Schematic illustrations of network types, (**b**) Original CLSM micrographs of appropriate examples (215 × 215 µm, 1024 × 1024 px).

More detailed, to identify the corresponding network type for a dough sample, the range of lacunarity should be determined first. Second, results of protein network analysis of the sample should be compared to its corresponding standard dough (without any additive) regarding to an increase or decrease of the values (c.f. arrows in Table 3). Hence, the corresponding network type, its interpretation, and its related dough rheological behaviour should have been identified. The strength of each microstructural or rheological attribute in general and in comparison to all network types is expressed by bold dots. Schematic illustrations of the network types as well as examples underline each network arrangement (c.f. Fig. 5).

When solely comparing the categorized strengths of network attributes (bold dots), it seems that the cleaved, particulate dense and particulate loose network types are quite similar (Table 3). However, the main difference between those three network types is the morphological characteristic lacunarity, which is very different for each type. Consequently, this results in completely different rheological properties of wheat dough. This example shows that sole evaluation of quantitative values of network attributes is challenging and that interpretation of the results is now supported due to the classification of network types, which allows conclusions on protein network arrangements and rheological behaviour of dough. In turn, this makes the mapping of different protein network types and similar rheological dough properties feasible and contributes therefore to the understanding of structure-function relationships. For example, a rigid as well as particulate, dense network can be linked to different protein network types.

Considering the nano- and microscale of gluten proteins, there are some network models combining structural properties with functionalities and product properties^37–41^. As this study shows that quantitative relations and predictions models can be performed between dough mechanical behavior and gluten microstructure, these findings can be used as a basis for future research. Hence, the network types can be linked to dough functionality with further investigations, especially using further rheological tests in the non-LVE, using large deformation extensional measurement, and under hyperbolic contraction flow^24,32,33^.

It should be noted, that the network types express distinctive forms of gluten arrangements and that gluten networks can also occur in an attenuated form of the six types. A standard dough can also be categorized into one of the six network types but to a lower extent. A standard dough had been described in literature as an interconnected gluten network covering starch granules^42^, a homogeneous protein phase with very fine structure^4^, or a spread network, which is defined as a continuous structure with elongated and distributed gluten strands^1^. These descriptions may be categorized to the network type “spread network”. However, Autio, *et al*.^34^ reported a looser protein network structure for a standard dough from an organic flour compared to regular flours. Comparing the descriptions of standard dough mentioned in literature, standard doughs might be categorized mostly to a spread or strengthened network as well as rarely to a cleaved or rigid network but to a lower extent. However, no universal standard dough exist in general due to variations in raw materials, different flour qualities and no standardized production process. Consequently, no standard protein network type exist and thus, no individual network type for a standard dough was established. Hence, individual standard doughs always need to be defined in relation to doughs within a measurement series or experiment.

## Conclusion

In this study, empirical relations of gluten microstructure and dough rheology were presented. Using specifically gluten-modified dough samples, linear (*J*_*el*_, *tanδ*, *z*), reciprocal (G*, *A*_*f*_) as well as logarithmic (*J*_*max*_, *J*_*r*_, *η*_0_) relations of dough rheology with gluten network attributes were found, and prediction models were defined by PLS regressions with an accuracy up to 90%. Furthermore, these prediction models provided reliable predicted rheological values for dough samples of low concentrations of unspecific gluten-modifying agents. Thus, with these mathematical models, it is possible to predict rheological properties via the five microstructural attributes lacunarity, branching rate, end-point rate, and average protein length as well as protein width. It should be noted that estimated linear models are based on values gained by micrographs of the specific magnification (60x objective) and resolution (215 × 215 µm) used in this study. Since microstructural results always depend on the applied setting and equipment, no absolute rheological values can be determined with prediction models in general. Nevertheless, comparative values can be determined with micrographs of other magnifications or resolutions, and estimations of rheological properties can be proposed with the models of the current study.

It was shown that prediction models could not be established for every kind of gluten network arrangements, especially for doughs effected by unspecific gluten-modifying agents due to additional impacts, like plasticizing or starch swelling effects. In spite of this, relations between dough rheology and gluten microstructure were determined based on cluster detections with principal component analysis. Hence, six network types were defined, which represent various distinctive gluten network arrangements. The definition of the network types is the first, which allows both quantitative definitions of gluten networks and the characterization of the relation of spatial arrangement of proteins to dough rheology. The classification of network types was designed to use it as a guiding scheme for the interpretation of protein network arrangements and prediction of corresponding dough rheological behaviour for further studies. Since rheological behaviour is related to bread quality, this study provides the basis for predictions and new approaches of structure-function relationships. Moreover, additional network types can be defined for more complex dough systems (e.g., with yeast or sodium chloride) or for doughs under mechanical forces (e.g., mixing, kneading, or sheeting) in the future. With the knowledge acquired in this study that quantitative relations and predictions between dough mechanical behaviour and gluten microstructure can be determined, further investigations and additional prediction models about dough functionality, processing behaviour and baking quality can be performed.

## Supplementary information

Supplementary Figure S1
