# Supplementary material for: Definition of network types – Prediction of dough mechanical behaviour under shear by gluten microstructure
Source: Sci Rep. 2019 Mar 18;9:4700. doi: 10.1038/s41598-019-41072-w (PMC6423116; doi:10.1038/s41598-019-41072-w)
Supplement: Supplementary file 1 — Supplementary Figure S1 [file 41598_2019_41072_MOESM1_ESM.pdf]

Definition of network types – Prediction of dough mechanical behaviour under shear by gluten microstructure

Isabelle Lucas, Hannes Petermeier, Thomas Becker, Mario Jekle

Supplementary data

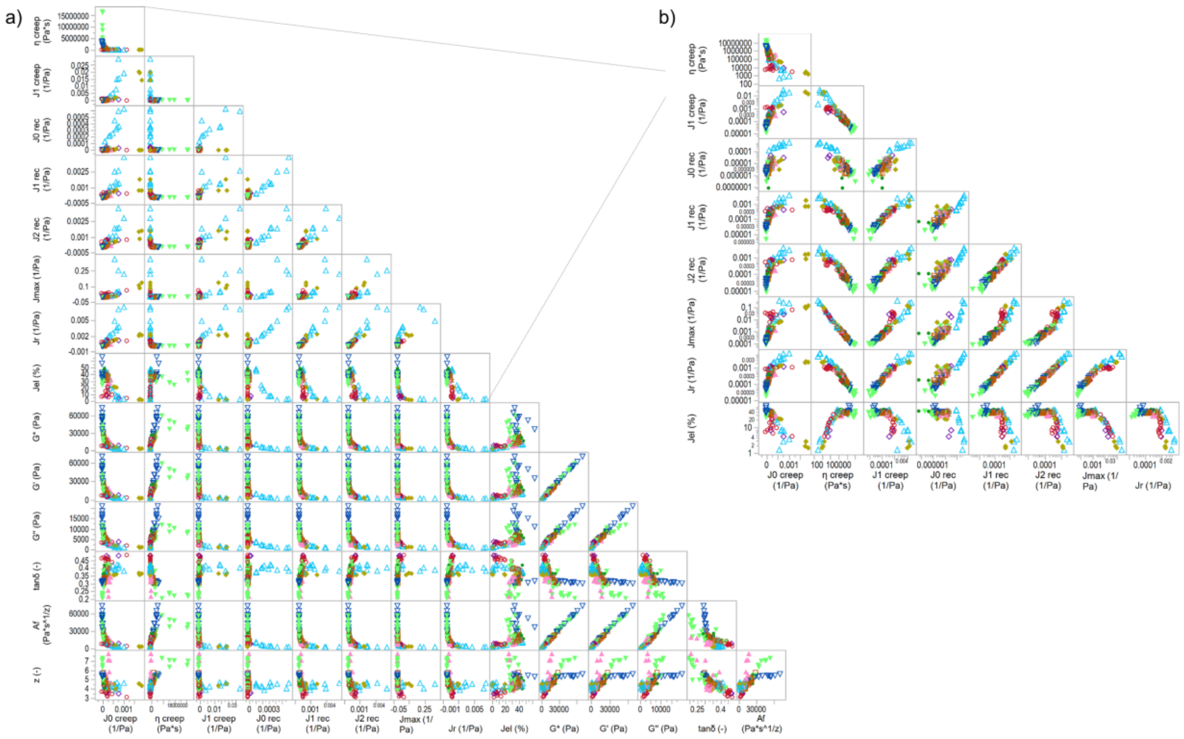

Supplementary Figure S1. Correlation matrix of the rheological data of unspecifically as well as specifically gluten-modified samples. (a) Correlation matrix of rheological attributes (oscillatory frequency test and creep-recovery test) of all gluten-modified samples. (b) Correlation matrix of attributes of creep-recovery test with logarithmic scales. Symbols: -●- ASC, -*- $KBrO_3$, -◊- BRN, -▲- SHO, -○- GSH, -□- GOX, -◆- ROI, -△- IHL, -▽- RHL, -▼- TG.
